# Ancestral reproductive bias in continuous-time branching trees under various sampling schemes

**DOI:** 10.1007/s00285-024-02105-9

**Published:** 2024-06-14

**Authors:** Jan Lukas Igelbrink, Jasper Ischebeck

**Affiliations:** 1 Institut für Mathematik, Goethe-Universität Frankfurt, Robert-Mayer-Str. 10, Frankfurt am Main, 60325 Hessen Germany; 2 Institut für Mathematik, Johannes Gutenberg-Universität Mainz, Staudingerweg 9, Mainz, 55128 Rheinland-Pfalz Germany

**Keywords:** Branching processes, Spines, Reproductive bias, Inspection paradox, Sampling schemes, Primary 60J80, Secondary 60K05, 92D10

## Abstract

Cheek and Johnston (JMB 86:70, 2023) consider a continuous-time Bienaymé-Galton-Watson tree conditioned on being alive at time *T*. They study the reproduction events along the ancestral lineage of an individual randomly sampled from all those alive at time *T*. We give a short proof of an extension of their main results (Cheek and Johnston in JMB 86:70, 2023, Theorems 2.3 and 2.4) to the more general case of Bellman-Harris processes. Our proof also sheds light onto the probabilistic structure of the rate of the reproduction events. A similar method will be applied to explain (i) the different ancestral reproduction bias appearing in work by Geiger (JAP 36:301–309, 1999) and (ii) the fact that the sampling rule considered by Chauvin et al. (SPA 39:117–130, 1991), (Theorem 1) leads to a time homogeneous process along the ancestral lineage.

## Introduction

Consider a continuous-time branching process with $N_t$ individuals alive at time *t*, started with one individual at time 0. At the end of its lifetime, an individual is replaced by a random number of independent offspring with distribution $(p_k)_{k\ge 0}$. When lifetimes of the individuals are i.i.d. with an arbitrary distribution $\mu$ on $\mathbb {R}_+$, the resulting process is called a *Bellman-Harris* process (Bellman and Harris 1948). In the special case of exponentially distributed lifetimes, this process is a continuous-time(Bienaymé-) Galton-Watson process, which is also called *one-dimensional continuous-time Markov branching process*, see (Athreya and Ney 1972, Chapter 3). For those processes, Cheek and Johnston (2023) study the process of reproduction times and family sizes along the ancestral lineage of an individual sampled from all those alive at a given time $T>0$, conditioned on the event $\{N_T>0\}$. We give a short and conceptual probabilistic proof of the main results of Cheek and Johnston (2023) in the more general Bellman-Harris setting. The core idea of this proof is as follows:

On the event $\left\{ N_T > 0 \right\}$, we assign to the individuals alive at time *T* independent random variables, which will be called markers, uniformly distributed on [0, 1]. Then the individual whose marker is largest constitutes a uniformly distributed random pick from all the individuals alive at time *T*. As we will see, the argument *s* of the generating functions that appear in the analytic arguments of Cheek and Johnston (2023) corresponds to the realisation of the largest marker. Sections 2–4 will be devoted to formulating and proving Theorem 1.

Relating to work of Chauvin et al. (1991), in Sect. 5 we will consider the case of potentially dependent but identically and atomless distributed markers and conditioning on one marker taking the prescribed value *s*. In contrast to the above, in this case one does not observe a time-inhomogeneity along the sampled ancestral lineage.

In Sect. 6 we will consider a planar embedding of the Bellman-Harris tree conditioned to survive up to time *T*, and analyse the leftmost ancestral lineage among those surviving until time *T*. Here we follow (Geiger 1999), who gave a representation of discrete-time Galton-Watson processes conditioned to survive up to a given number of generations. With this sampling rule we observe a time-inhomogeneity of ancestral reproduction events that is different from the one in Cheek and Johnston (2023).

In Sect. 7 we briefly resume the discussion from Cheek and Johnston (2023) on a possible relation between the ancestral rate bias and the rate of mutations per cell division in embryogenesis, and illustrate the various sampling schemes from a more biological perspective.

## Sampling an ancestral line at random

**Fig. 1:**
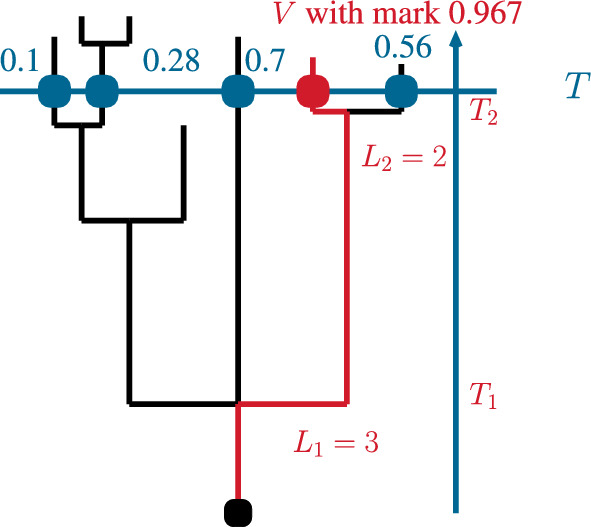
An example for a realisation of the random variables $S, L_1, L_2, T_1, T_2$ in the sampling regime described in Sect. 2

Recall that to each individual at time *T*, we have associated a uniform marker in [0, 1]. On the event $\{N_T > 0\}$, let the individual *V* be sampled as described in the Introduction, and let *S* be its mark. We define the process $(N_t)_{t\ge 0}$ to be right continuous with left limits. As a consequence, if $T_1$ is the lifetime of the root individual, then $N_{T_1}$ has distribution $\left( p_k\right) _{k\ge 0}$. Let *J* be the random number of reproduction events and $0<T_1< T_2< \cdots < T_J\le T$ be the random times of reproduction events along the ancestral lineage of *V*. Let $L_1, \ldots , L_J$ be the offspring sizes in these reproduction events and let $0<\tau _1< \tau _2 < \cdots$ be the random arrival times in a renewal process with interarrival time distribution $\mu$. See Fig. 1 for a sample realisation.

Denote by ${{\,\mathrm{\textbf{P}}\,}}$ and ${{\,\mathrm{\textbf{E}}\,}}$ the probability measure and expectation for $N_0=1$.

### Theorem 1

For $j \ge 0$, $0< t_1<\ldots < t_j \le T \in \mathbb {R}$ and $\ell _1,\ldots , \ell _j \in \mathbb {N}$ we have

1 $$\begin{aligned}&{{\,\mathrm{\textbf{P}}\,}}\left( N_T>0, J=j, \, T_1\in \textrm{d}t_1, \ldots T_j\in \textrm{d}t_j,\, L_1=\ell _1, \ldots , L_j=\ell _j,\, S\in \textrm{d}s\right) \nonumber \\&\quad = {{\,\mathrm{\textbf{P}}\,}}\left( \tau _1 \in \textrm{d}t_1, \ldots , \tau _j \in \textrm{d}t_j, \tau _{j+1} > T\right) \prod _{i=1}^j \left( \ell _i p_{\ell _i} {{\,\mathrm{\textbf{E}}\,}}\left[ s^{ N_{T-t_i} } \right] ^{\ell _i -1 } \right) \textrm{d}{s}. \end{aligned}$$

### Corollary 2

When integrated over $s\in (0,1)$, (1) reveals that the process

$(T_1,L_1),\ldots , (T_J,L_J)$ of reproduction times and offspring sizes along the ancestral lineage of the uniformly chosen individual (conditioned on $\{N_T>0\}$) is a mixture of (what could be called) “biased compound renewal processes”.

When the lifetime distribution $\mu$ is the exponential distribution with parameter *r*, then $\tau _1, \tau _2,\ldots$ are the points of a rate *r* Poisson point process. In this case Corollary 2 together with (1) becomes a reformulation of the statements of (Cheek and Johnston 2023, Theorems 2.3 and 2.4), and at the same time reveals the probabilistic role of the mixing parameter *s* in the mixture of biased compound Poisson processes that appear in the “Cox process representation” of Cheek and Johnston (2023).

Let us write (as in Cheek and Johnston (2023)) $F_t(s):= \textbf{E}[s^{N_t}]$, and abbreviate

2 $$\begin{aligned} B(t,T, \ell ):= \frac{1}{1-F_T(0)} \int _0^1 F_{T-t}(s)^{\ell -1}F'_T(s) \textrm{d}s. \end{aligned}$$

Cheek and Johnston (2023), (Theorem 2.4) (as well as Theorem 1) says that the rate of size $\ell$ reproduction along the uniform ancestral lineage at time *t* is

$$r\ell p_\ell \, B(t,T,\ell ).$$

This can be obtained from Corollary 2 by noting that *S* has density

$$\begin{aligned} \displaystyle \frac{F_T'(s)}{1-F_t(0)}. \end{aligned}$$

In this sense the factor $B(t,T,\ell )$ can be interpreted as an *(ancestral) rate bias*, on top of the classical term $r \ell p_\ell$. Indeed, the factor $B(t,T,\ell )$ is absent in trees that are biased with respect to their size at time *T*. Galton-Watson trees of this kind have been investigated (also in the multitype case) by (Georgii and Baake 2003, Sect. 4); they are continuous-time analogues of the size-biased trees analysed by Lyons et al. (1995) and Kurtz et al. (1997).

In the critical and supercritical case one can check that, for all fixed $u<T$ and $\ell \in \mathbb {N}$ one has the convergence $B(T-u, T, \ell ) \rightarrow 1$ as $T\rightarrow \infty$ because *S* converges to 1 in probability. In the supercritical case this stabilisation along the sampled ancestral lineage corresponds to the“retrospective viewpoint” that has been taken in Georgii and Baake (2003) and, in the more general situation of Crump-Mode-Jagers processes, by Jagers and Nerman (1996). The choice $\mu = \delta _1$ renders the case of discrete-time Galton-Watson processes, starting with one individual at time 0 and with reproduction events at times $1,2,\ldots$. Then, with $T=n \in \mathbb {N}$, and $L_1, \ldots , L_{n}$ being the family sizes along the ancestral lineage of the sampled individual *V*, the formula (1) specialises to

3 $$\begin{aligned} {{\,\mathrm{\textbf{P}}\,}}\left( N_n>0, \, L_1=\ell _1, \ldots , L_{n}=\ell _{n},\, S\in \textrm{d}s\right) = \left( \prod _{i=1}^{n} \ell _i p_{\ell _i} {{\,\mathrm{\textbf{E}}\,}}\left[ s^{ N_{n-i} } \right] ^{\ell _i -1 } \right) \textrm{d}{s}. \end{aligned}$$

## Maxima of i.i.d. random markers

As a preparation for the short probabilistic proof of Theorem 1 given in the next section, we recall the following well-know fact: Denote by Unif[0, 1] the uniform distribution on the interval [0, 1]. For $\ell \in \mathbb {N}$, let $\widetilde{S}$ be the maximum of $\ell$ independent Unif[0, 1]-distributed random variables $U_1, \ldots , U_\ell$. Then the density of $\widetilde{S}$ is

4 $$\begin{aligned} {{\,\mathrm{\textbf{P}}\,}}\left( \widetilde{S} \in \textrm{d}s\right) = \ell s^{\ell -1} \textrm{d}s, \quad 0\le s \le 1. \end{aligned}$$

Indeed, because of exchangeability,

$${{\,\mathrm{\textbf{P}}\,}}\left( \widetilde{S}\in \textrm{d}s\right) = \ell {{\,\mathrm{\textbf{P}}\,}}\left( U_1\in \textrm{d}s\right) {{\,\mathrm{\textbf{P}}\,}}\left( U_2<s,\cdots , U_\ell <s\right) ,$$

which equals the r.h.s. of (4).

The following lemma specialises to (4) when putting $\tilde{N} \equiv 1$.

### Lemma 3

Let $\widetilde{N}$ be an $\mathbb {N}_0$-valued random variable, and $\widetilde{N}_1, \widetilde{N}_2, \ldots$ be i.i.d. copies of $\tilde{N}$. Given $\widetilde{N}_1, \widetilde{N}_2, \ldots$ let $U_{1,1}, \ldots U_{1,\widetilde{N}_1}, U_{2,1}, \ldots U_{2,\widetilde{N}_2}, \ldots$ be independent Unif [0, 1]-distributed random variables, and write

$$\begin{aligned} S_k:= & {} \max \left\{ U_{k,1}, \ldots , U_{k,\widetilde{N}_k}\right\} , \quad k=1,2,\ldots \\ S^{(\ell )}:= & {} \max \left\{ S_1, \ldots , S_\ell \right\} ,\quad \ell \in \mathbb {N}\end{aligned}$$

where we put $\max (\emptyset ):= -\infty$. Then, for all $\ell \in \mathbb {N}$, the density of $S^{(\ell )}$ is

5 $$\begin{aligned}{} & {} {{\,\mathrm{\textbf{P}}\,}}\left( \widetilde{N}_1+\ldots +\widetilde{N}_\ell> 0, \, S^{(\ell )} \in \textrm{d}s\right) \nonumber \\{} & {} \qquad = \ell \, {{\,\mathrm{\textbf{E}}\,}}\left[ s^{\widetilde{N}}\right] ^{\ell -1} {{\,\mathrm{\textbf{P}}\,}}\left( \widetilde{N}_1 > 0, \,S_1 \in \textrm{d}s\right) , \quad 0\le s \le 1. \end{aligned}$$

### Proof

Again because of exchangeability, the l.h.s. of (5) equals

6 $$\begin{aligned} \ell {{\,\mathrm{\textbf{P}}\,}}\left( \widetilde{N}_1 > 0, \, S_1 \in \textrm{d}s\right) {{\,\mathrm{\textbf{P}}\,}}\left( S_2< s, \ldots , S_\ell < s\right) \end{aligned}$$

for $$s\in [0,1]$$. Since by assumption the $$S_k$$ are i.i.d. copies of $$ S_1$$, the rightmost factor in (6) equals

$${{\,\mathrm{\textbf{P}}\,}}\left( S_1< s\right) ^{\ell -1} = {{\,\mathrm{\textbf{E}}\,}}\left[ {{\,\mathrm{\textbf{P}}\,}}\left( S_1 < s \mid \widetilde{N}_1\right) \right] ^{\ell -1} = {{\,\mathrm{\textbf{E}}\,}}\left[ s^{\widetilde{N}_1}\right] ^{\ell -1} = {{\,\mathrm{\textbf{E}}\,}}\left[ s^{\widetilde{N}}\right] ^{\ell -1}.$$

Hence, (6) equals the r.h.s. of (5), completing the proof of the lemma. $\square$

The following corollary is immediate.

### Corollary 4

Let *L* be an $\mathbb {N}_0$-valued random variable that is independent of all the random variables appearing in Lemma 3, with ${{\,\mathrm{\textbf{P}}\,}}(L=\ell ) = p_\ell$, $\ell \in \mathbb {N}_0$. Then we have for all $\ell \in \mathbb {N}_0, 0\le s \le 1$,

7 $$\begin{aligned} {{\,\mathrm{\textbf{P}}\,}}\left( L=\ell ,\, \widetilde{N}_1+\cdots +\widetilde{N}_\ell> 0, \, S^{(\ell )} \in \textrm{d}s\right) = \ell p_\ell \, {{\,\mathrm{\textbf{E}}\,}}\left[ s^{\widetilde{N}}\right] ^{\ell -1} {{\,\mathrm{\textbf{P}}\,}}\left( \widetilde{N}_1 > 0, \,S_1 \in \textrm{d}s\right) .\nonumber \\ \end{aligned}$$

## Proof of Theorem 1

We prove the statement (1) by induction over *j*, *simultaneously* over all time horizons $T > 0$. We write $\textbf{P}^T$ for the probability referring to time horizon *T*; this will be helpful in the induction step where we will encounter two different time horizons.

For $j=0$, both sides of (1) are equal to $\mu ((T,\infty ))\, \textrm{d}s$.

For $j=1$, on the event $\{T_1\in \textrm{d}{t_1}\}$, we can directly apply Corollary 4 to the markers of the $L_1$ subtrees produced in this event. These subtrees live $T-t_1$ long and thus have sizes distributed as $N_{T-t_1}$. So the left side of (1) equals

$$\begin{aligned} {{\,\mathrm{\textbf{P}}\,}}\left( \tau _1 \in \textrm{d}{t_1}\right) p_{\ell _1} \ell _1 {{\,\mathrm{\textbf{E}}\,}}\left[ s^{N_{T-t_1}} \right] ^{\ell _1-1} {{\,\mathrm{\textbf{P}}\,}}^{T-t_1} \left( T_1 > T-t_1, \, S\in \textrm{d}{s} \right) , \end{aligned}$$

which is using the $j=0$ case. This is equal to the right hand side of (1).

Now assume we have proved (1) for all time horizons $T'$ with $j-1$ (in place of *j*), for all times $t_1', \dots , t_{j-1}'\le T'$, sizes $\ell _1', \dots , \ell _{j-1}' \in \mathbb {N}$ and $s\in [0,1]$. On the event $\left\{ T_1 \in \textrm{d}t_1, L_1 = \ell _1 \right\}$ the descendants of the $\ell _1$ siblings in the first branching event form $\ell _1$ independent and identically distributed trees on the time interval $[t_1, T]$. Thus, using Corollary 4 and setting $t_1':= t_2-t_1, \ldots , t_{j-1}'= t_j-t_{1}$, we obtain that the left hand side of (1) equals

8 $$\begin{aligned} {{\,\mathrm{\textbf{P}}\,}}&\left( \tau _1 \in \textrm{d}{t_1}\right) p_{\ell _1} \ell _1 {{\,\mathrm{\textbf{E}}\,}}\left[ s^{N_{T-t_1}} \right] ^{\ell _1-1} \nonumber \\ {}&\cdot {{\,\mathrm{\textbf{P}}\,}}^{T-t_1} \left( J=j-1, \, T_1 \in \textrm{d}{t_1}', \ldots , T_{j-1} \in \textrm{d}{t_{j-1}}', \, N_{T-t_1} >0, \, S\in \textrm{d}{s} \right) . \end{aligned}$$

By the induction assumption, this is equal to

9 $$\begin{aligned} {{\,\mathrm{\textbf{P}}\,}}&\left( \tau _1 \in \textrm{d}{t_1} \right) p_{\ell _1} \ell _1 \nonumber \\&\cdot {{\,\mathrm{\textbf{P}}\,}}\left( \tau _1' \in \textrm{d}{t_1}', \ldots , \tau _{j-1}' \in \textrm{d}{t_{j-1}'}, \, \tau _j' \ge T-t_1 \right) \prod _{i=2}^j \left( \ell _i p_{\ell _i} {{\,\mathrm{\textbf{E}}\,}}\left[ s^{N_{T-t_i}} \right] ^{\ell _i-1} \right) , \end{aligned}$$

where $\left( \tau _1', \tau _2', \ldots \right)$ have the same distribution as $\left( \tau _1, \tau _2,\ldots \right) .$ Obviously (9) equals the r.h.s of (1). This completes the induction step and concludes the proof. $\square$

## Conditioning on a marker value

Chauvin et al. (1991) consider a Markov process with an atomless transition probability indexed by a continuous-time Galton-Watson-tree and they then condition on an individual at time *T* to be at a given location.

To relate this to the framework described in the Introduction, we assume that each individual alive at time *T* in the Bellmann-Harris tree carries a marker in some standard Borel space *E* and these random marks have the following properties:

1. Their marginal distributions (denoted by $\nu$) are identical and do not depend on the reproduction events
2. A.s. no pair of marks is equal.

Think for example of branching Brownian motion: The positions of the different particles clearly depend on each other via the genealogy, however, at time *t* the marginal distribution of the position of each particle is a centered Gaussian random variable with variance *t*, irrespective of its past genealogical events in the underlying continuous-time Galton-Watson tree. Thus (M1), is fulfilled. Since two correlated Gaussian random variables are a.s. not equal if the correlation coefficient is not equal to one, (M2) is also fulfilled.

**Fig. 2:**
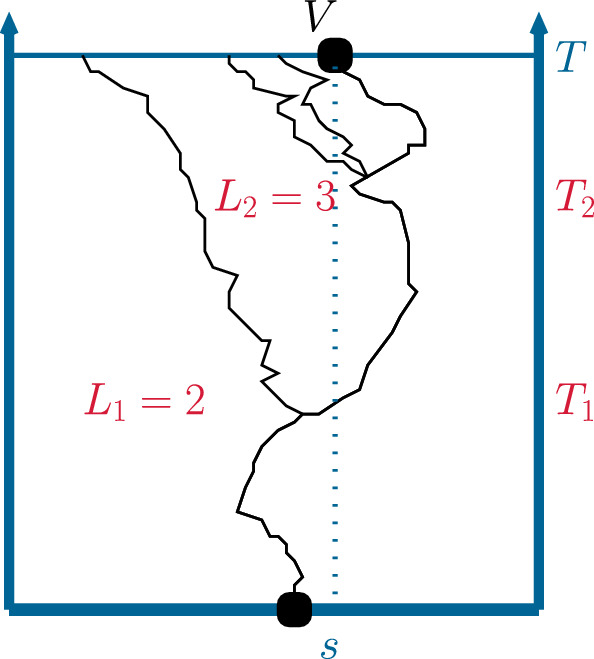
An example for a realisation of the random variables $L_1, L_2, T_1, T_2$ in the sampling regime described in Sect. 5

We now condition on $\left\{ N_T>0\right\}$ and, for given $s\in E$, on one of the $N_T$ individuals having marker value *s*. Remember the previous notation: Denote by *V* the individual having marker *s*. Let *J* be the random number of reproduction events along the ancestral lineage of *V* and $0<T_1< T_2< \cdots< T_J< T$ be the random times of these reproduction events. Let $L_1, \ldots , L_J$ be the offspring sizes in these reproduction events and let $0<\tau _1< \tau _2 < \cdots$ be the random arrival times in a renewal process with interarrival time distribution $\mu$. Figure 2 depicts a sample realisation.

The following Theorem generalises (part of) (Chauvin et al. 1991, Theorem 2) to general lifetime time distributions.

### Theorem 5

For $j \ge 0$, $0< t_1<\ldots< t_j < T$ and $\ell _1,\ldots , \ell _j \in \mathbb {N}$ we have for $\nu$-almost all *s*

10 $$\begin{aligned}&{{\,\mathrm{\textbf{P}}\,}}\left( \left. J=j, \, T_1\in \textrm{d}t_1, \ldots , T_j\in \textrm{d}t_j,\, L_1=\ell _1, \ldots , L_j=\ell _j \right| N_T>0, \exists \, \textrm{ marker }\in \textrm{d}s \right) \nonumber \\&\qquad = \frac{1}{{{\,\mathrm{\textbf{E}}\,}}[N_T]}{{\,\mathrm{\textbf{P}}\,}}\left( \tau _1 \in \textrm{d}t_1, \ldots , \tau _j \in \textrm{d}t_j, \tau _{j+1} \ge T\right) \prod _{i=1}^j \ell _{i} p_{\ell _i}. \end{aligned}$$

### Proof

Because of properties (M1), (M2) we have

$${{\,\mathrm{\textbf{P}}\,}}(N_T > 0, \exists \, \textrm{ marker }\in \textrm{d}s) = {{\,\mathrm{\textbf{E}}\,}}[N_T]\nu (\textrm{d}s), \quad s \in E.$$

Hence (10) is equivalent to

11 $$\begin{aligned}&{{\,\mathrm{\textbf{P}}\,}}\left( J=j, \, T_1\in \textrm{d}t_1, \ldots , T_j\in \textrm{d}t_j,\, L_1=\ell _1, \ldots , L_j=\ell _j, N_T>0, \exists \, \textrm{ marker }\in \textrm{d}s \right) \nonumber \\&\qquad ={{\,\mathrm{\textbf{P}}\,}}\left( \tau _1 \in \textrm{d}t_1, \ldots , \tau _j \in \textrm{d}t_j, \tau _{j+1} \ge T\right) \prod _{i=1}^j \ell _{i} p_{\ell _i} \, \nu (\textrm{d}s). \end{aligned}$$

As in the proof of Theorem 1 we prove the statement (11) by induction over *j*, *simultaneously* over all time horizons $T > 0$. As before we write $\textbf{P}^T$ for the probability referring to time horizon *T*. For $j=0$ the statement is true, since

$${{\,\mathrm{\textbf{P}}\,}}^T(J=0, N_T>0, \exists \, \textrm{ marker }\in \textrm{d}s) = {{\,\mathrm{\textbf{P}}\,}}\left( \tau _1 \le T\right) \, \nu (ds).$$

Assume we have proved (11) for all time horizons $T'$ with $j-1$ (in place of *j*), for all times $t_1', \dots , t_{j-1}'\le T'$, sizes $\ell _1', \dots , \ell _{j-1}' \in \mathbb {N}$ and marker distributions with the same marginal $\nu$ that satisfy conditions (M1), (M2). Turning to (11) as it stands, we note that on $\{T_1 = t_1, L_1=\ell _1\}$, the descendants of the $\ell _1$ siblings in the first branching event form $\ell _1$ independent and identically distributed trees on the time interval $[t_1, T]$. Let $\mathcal {U}_k,\, k=1,\dots , \ell _1$, be the set of markers of the individuals at time *T* that descend from the *k*-th sibling. By randomly permuting these $\ell _1$ siblings, we can assume that the set-valued random variables $\mathcal {U}_k,\,k=1,\ldots , \ell _1$, are exchangeable. Note that the markers in each $\mathcal {U}_k$ satisfy conditions (M1), (M2). Because the markers are a.s. pairwise different by assumption, the marker *s* belongs to at most one of those $\mathcal {U}_k$, so

12 $$\begin{aligned} \textbf{1}_{\{ \exists \, \textrm{ marker }\,\in \, \textrm{d}s \}} = \sum _{k=1}^{\ell _1} \textbf{1}_{\{\mathcal {U}_k \,\cap \, \textrm{d}s\,\ne \, \emptyset \}} \, \text{ a.s. } \end{aligned}$$

Note that for the sake of intuition we use a differential notation for what formally is an (integral) equality for the distribution of the random point measure formed by the individuals’ markers, which by assumption (M2) can be seen as a random set of points.

Putting $t_1':= t_2-t_1, \ldots , t_{j-1}':= t_j-t_1$ we thus infer, using the branching property of the Bellman-Harris tree, that the left hand side of (11) equals

13 $$\begin{aligned} \begin{aligned} {{\,\mathrm{\textbf{P}}\,}}(\tau _1 \in \textrm{d}t_1)p_{\ell _1} \ell _1 \cdot {{\,\mathrm{\textbf{P}}\,}}^{T-t_1}\Bigl (&J=j-1, \,T_1 \in \textrm{d}t_1',\ldots , T_{j-1} \in \textrm{d}t_{j-1}',\\&L_1=\ell _2,\ldots , L_{j-1}=\ell _j, N_{T-t_1} > 0, \exists \, \textrm{mark} \in \textrm{d}s\Bigr ). \end{aligned}\nonumber \\ \end{aligned}$$

By the induction assumption this is equal to

14 $$\begin{aligned} {{\,\mathrm{\textbf{P}}\,}}(\tau _1 \in \textrm{d}t_1)p_{\ell _1} \ell _1{{\,\mathrm{\textbf{P}}\,}}\left( \tau _1' \in \textrm{d}t_1', \ldots , \tau _{j-1}' \in \textrm{d}t_{j-1}', \tau _{j}' \ge T-t_1\right) \prod _{i=2}^j \ell _{i} p_{\ell _i} \, \nu (\textrm{d}s),\nonumber \\ \end{aligned}$$

where $(\tau _1', \tau _2', \ldots )$ have the same distribution as $(\tau _1, \tau _2,\ldots )$. Obviously (14) equals the r.h.s. of (11), which completes the induction step and concludes the proof. $\square$

### Remark 1

If $\mu$ is the exponential distribution with parameter *r*, then $\tau _1, \tau _2, \ldots$ are again the points of a rate *r* Poisson point process and (10) implies that reproduction events along the ancestral lineage of *V* happen according to a time-homogeneous Poisson process with rate $r \sum _{\ell }\ell p_{\ell }$. This corresponds to the description of the events along the ancestral line of *V* given in (Chauvin et al. 1991, Theorem 1).

## Sampling the left-most ancestral lineage

**Fig. 3:**
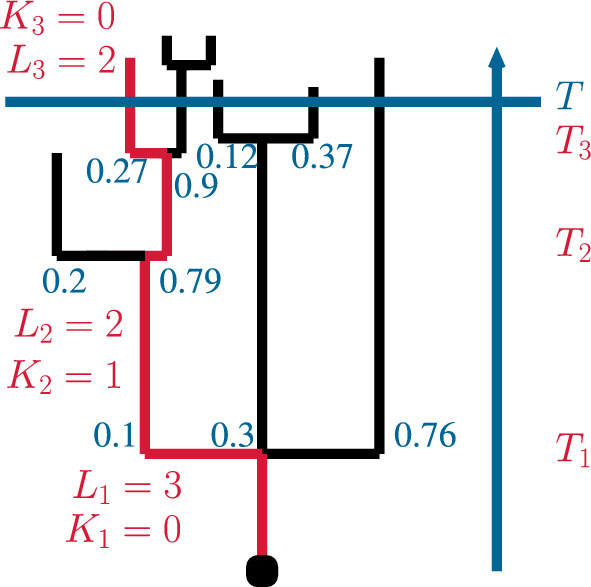
An example for a realisation of markers and random variables $L_1, L_2,K_1, k_2, T_1, T_2$ in the sampling regime described in Sect. 6

We now aim to obtain results about what Geiger (1999) calls the leftmost surviving ancestral lineage in a planar embedding of the tree: At any reproduction event we assign independent uniformly on [0, 1] distributed markers to all children. An individual can now be uniquely determined by the markers along its ancestral lineage. On the event $\{N_T > 0\}$, let *V* be the individual whose markers along the entire ancestral lineage comes first in the lexicographic ordering. Let *J* be the random number of reproduction events and $0< T_1< T_2< \cdots < T_J \le T$ be the random times of reproduction events along the ancestral lineage of *V*. Let $L_1, \ldots , L_J$ be the offspring sizes in these reproduction events and let $0<\tau _1< \tau _2 < \cdots$ be the random arrival times in a renewal process with interarrival time distribution $\mu$. Denote by $K_i$ the number of siblings born at reproduction event number *i* along the ancestral lineage of *V* which have a lower lexicographic order than *V* and whose descendants hence die out before time *T*. Figure 3 shows a realisation for this sampling rule.

### Theorem 6

For $j \ge 0$, $0< t_1<\ldots< t_j < T, \, \ell _1,\ldots , \ell _j \in \mathbb {N}$ and $k_i \in \left\{ 1,\ldots , \ell _i-1 \right\}$ we have

15 $$\begin{aligned}&{{\,\mathrm{\textbf{P}}\,}}\left( N_T>0, J=j, \, T_1\in \textrm{d}t_1, \ldots , T_j\in \textrm{d}t_j,\, L_1=\ell _1, \ldots , L_j=\ell _j, K_1=k_1,\ldots , K_j=k_j\right) \nonumber \\&\quad = {{\,\mathrm{\textbf{P}}\,}}\left( \tau _1 \in \textrm{d}t_1, \ldots , \tau _j \in \textrm{d}t_j, \tau _{j+1} \ge T\right) \prod _{i=1}^j \left( p_{\ell _i}{{\,\mathrm{\textbf{P}}\,}}\left( N_{T-t_i} =0\right) ^{k_i} \right) . \end{aligned}$$

### Proof

The proof of the theorem works in analogy to the one of Theorem 1, but using following analogue of Lemma 3. $\square$

### Lemma 7

Let $\widetilde{N}$ be an $\mathbb {N}_0$-valued random variable, and $\widetilde{N}_1, \widetilde{N}_2, \ldots$ be i.i.d. copies of $\tilde{N}$. Given $\widetilde{N}_1, \widetilde{N}_2, \ldots$ let $U_{1}, U_{2}, \ldots$ be independent Unif [0, 1]-distributed random variables, and write

$$\begin{aligned} S^{(\ell )}:= & {} \min \left\{ U_{k} \mid \widetilde{N}_k \ge 1, k=1,\ldots ,\ell \right\} , \\ K^{(\ell )}:= & {} \left| \left\{ U_k \mid U_k < S^{(\ell )}, k=1,\ldots ,\ell \right\} \right| \end{aligned}$$

where we put $\min (\emptyset ):= {+}\infty$. Then, for all $k < \ell \in \mathbb {N}$ we have

16 $$\begin{aligned} {{\,\mathrm{\textbf{P}}\,}}\left( \widetilde{N}_1+\ldots +\widetilde{N}_\ell> 0, \, K^{(\ell )} = k \right) = {{\,\mathrm{\textbf{P}}\,}}\left( \widetilde{N}=0\right) ^{k} {{\,\mathrm{\textbf{P}}\,}}\left( \widetilde{N} > 0\right) . \end{aligned}$$

### Proof

Because $S^{(\ell )}$ and $K^{(\ell )}$ do not depend on the order of $U_1, \dots , U_\ell$, we can use exchangeability to assume that $U_1<U_2<\dots <U_\ell$. For $K^{(\ell )}$ to be *k*, $S^{(\ell )}$ has then to be $U_{k+1}$. This is exactly the case if $\widetilde{N}_1, \dots , \widetilde{N}_k = 0$ and $\widetilde{N}_{k+1}>0$. $\square$

## Biological perspectives

Cheek and Johnston (2023, Sect. 5) discuss recent studies (Park et al. (2021), Coorens et al. (2019)) which suggest that certain mutation rates are elevated for the earliest cell divisions in embryogenesis. Under the assumptions that (1) cell division times vary and (2) mutations arise not only *at* but also *between* cell divisions, Cheek and Johnston argue that this early rate elevation might be parsimoniously explained by their finding that in the supercritical case with no deaths the rate of branching events along a uniformly chosen ancestral lineage is increasing in $t \in [0,T]$ (which is a corollary to their Theorem 2.4).

The two-stage sampling rule

- first sample a random tree (“an adult”) that survives up to time *T*,
- then sample an individual from this tree (“a cell from this adult”) at time *T*

seems adequate for the situation discussed in (Cheek and Johnston 2023, Sect. 5). In other modeling situations, again with a large collection of i.i.d. Galton-Watson trees, one may think of a different sampling rule: Choose individuals at time *T* uniformly from the union of all time *T* individuals in all of the trees. This makes it more probable that the sampled individuals belong to larger trees, and in fact corresponds to the size-biasing of the random trees at time *T* (Georgii and Baake 2003, Sect. 4). In the two-stage sampling rule we see the different rate bias (2), discussed at the end of Sect. 2.

As can be seen from (Chauvin et al. 1991, Theorem 1) (and Theorem 5), the rate bias (2) is also absent along the ancestral lineage of an individual whose marker has a prescribed value *s*, if one considers a situation in which a neutral marker evolves along the trees in small (continuous) mutation steps, and if one takes, for the prescribed value *s*, the collection of trees so large that one individual at time *T* has a marker value close to (ideally: precisely at) *s*.

The sampling rule that appears in Geiger (1999) (and Theorem 6) leads to a rate (and reproduction size) bias along the ancestral lineage that is different from the ones we just discussed. This sampling rule can be defined via i.i.d. real-valued valued neutral markers that are created at each birth and passed to the offspring. The individual sampled at time *T* (from the tree conditioned to survive up to time *T*) is the one whose marker sequence is the largest in lexicographic order among the individuals that live in the tree at time *T*. This interpretation appears of less biological relevance, except in the pure birth (or cell division) case, where one might think of one single marker that is passed on in each generation to a randomly chosen daughter cell.

## Data Availability

We do not analyse or generate any datasets, because our work proceeds within a theoretical and mathematical approach.
